# Vaginal delivery through annular placenta – case report

**DOI:** 10.3325/cmj.2013.54.203

**Published:** 2013-04

**Authors:** Nikica Živković, Stipe Krezo, Ratko Matijević, Krešimir Živković

**Affiliations:** 1 Department of obstetrics and gynecology, Šibenik General Hospital, Šibenik, Croatia; 2 Department of obstetrics and gynecology, “Hrvatski Ponos” General Hospital, Knin, Croatia; 3 University Department of Obstetrics and Gynecology, “Sveti Duh” University Hospital, Zagreb University School of Medicine, Zagreb, Croatia

## Abstract

Annular placenta is an extremely rare morphological type of human placenta. It is commonly related to placental vessel abnormalities frequently causing antenatal and postnatal hemorrhage and operative delivery. Gravida 4 para 1 had an uneventful course of pregnancy and normal vaginal delivery followed by moderate postpartum hemorrhage. Hemorrhage was found to be local in origin but the placenta was annular in shape and the newborn was delivered through one of the openings. Annular placenta was not recognized before delivery. Its implantation site was in the lower uterine segment but high enough to allow the passage of the fetus through its annular defect and vaginal birth. To our knowledge, this is a first report of annular placenta ending in normal vaginal delivery.

The definitive shape of the placenta is usually determined by the initial distribution of the villi over the chorionic surface (1). Annular placenta is a placenta in the form of a band encircling the interior of the uterus. It is very rare but commonly related to placental vessel abnormalities, frequently causing antenatal and postnatal hemorrhage and operative delivery (2). In this report, we describe a case of annular placenta ending in normal vaginal delivery.

## Case

A 35-year old gravida 4 para 1 was admitted to the labor ward of Šibenik General Hospital, Šibenik, Croatia, in the early stage of labor at 39 + 3 weeks. She already had one vaginal birth 7 years ago and two early pregnancy losses (incomplete miscarriage), which both ended with instrumental evacuation of the uterus. The course of her present pregnancy was uneventful and she received her antenatal care in an outpatient unit. There was no medical or family history information relevant for her pregnancy. She had nine antenatal visits and three ultrasound examinations during pregnancy. All findings were normal and the placental position was reported to be on the posterior uterine wall.

On admission, she was well, her blood pressure and pulse rate were within reference ranges, cardiotocography (CTG) recording was normal and reactive with irregular contractions. Her uterus was soft, with no pain or tenderness; the baby was in cephalic position and the head was 4/5 palpable on abdominal examination. On vaginal examination, the cervix was fully effaced and 4 cm dilated. Through membranes, the fetal head was felt above the level of the spines. The diagnosis of the latent first stage of labor was made and she was kept on the labor ward with intermittent cardiotocography (CTG) monitoring, mostly because she lived in rural area far away from the hospital. There was no intervention in the sense of active management on the woman’s request. She was mobilized and she did not require analgesia.

The following morning, the contractions stopped. CTG was normal and reactive. At that time, she was 8 cm dilated and after having given informed consent she opted for amniotomy and augmentation of labor. Amniotic fluid was normal and iv. infusion of oxytocine was started with infusion rate of 0.5 mU/min, increasing in 15-minute intervals until regular contractions. One hour later she was contracting 1:3 minutes apart with normal reactive CTG. Two hours after amniotomy, she had an episode of fresh vaginal bleeding. There was no obvious source, it was not heavy, and was related to cervical dilatation and head engagement. The bleeding stopped without intervention. CTG was reactive. One hour later she gave birth to a normal live female newborn weighting 3700 g. The Apgar score after one and 5 minutes was 10.

**Figure 1:**
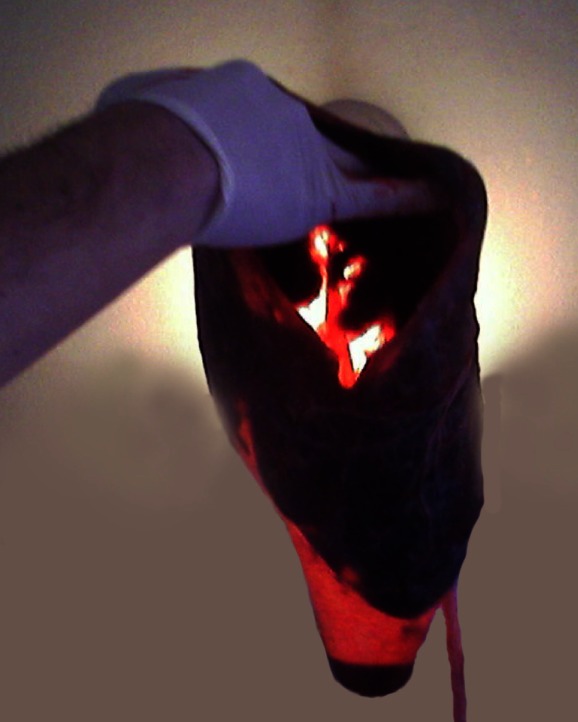
Defect on one side of annular placenta through which the child passed during the delivery (where the hand is placed) and intact amniotic membrane remaining on the opposite side

Immediately after delivery, she started to bleed heavily. Full blood count and clotting screen was taken, and the two units were cross-matched to maternal blood. The placenta was delivered by continuous cord traction and found to be annular with intact membranes on the one side and with a hole on the other, with torn membranes on its edges (Figure 1). Bimanual compression was applied to the uterus, infusion rate of oxytocin was increased, and ergometrine 0.5 mg was given i.v., followed by exploration of the uterus and birth canal under general anesthesia. The uterus was found to be empty and bleeding was located on the cervical tear extending 7 cm and was controlled by polyglactin sutures. After suturing, the bleeding stopped, a good tonus of the uterus was obtained, and medio-lateral episiotomy was sutured in a routine manner. She was under close surveillance in the 4th stage of labor, anemia (Hb value 88 g/L) was corrected with two units of blood on the second day, and she was discharged home on the day four.

Following a close inspection, the placenta was found to lack the central part of tissue on both sides. There was a defect on one side, through which the child passed during the delivery, with an intact amniotic membrane remaining on the opposite side (Figure 1). The diagnosis of annular placenta was made. The insertion of the umbilical cord was paracentral.

## Comment

Annular placenta is likely to be derived from placenta previa with focal atrophy of the low-lying villous tissue covering the internal os (3). We believe that multiparity and cervical insufficiency was likely to be one of supporting factors in this case, as there was a “free space” above the internal os, where trophoblastic invasion did not occur. Based on that, annular placenta can be diagnosed by ultrasound (2), which was missed in this case. We believe that the placenta was correctly visualized on level-one scan, but its presence all around the uterine cavity was not recognized. Its position was not fulfilling the criteria of either placenta previa or low-lying placenta (was not within 2 cm of the internal os) and therefore further investigation was not indicated. What makes our case unusual is that annular placenta was not diagnosed as placenta previa before the delivery. Ultrasound did not report placenta previa, on admission there was no placental tissue felt on vaginal examination, the CTG was normal, the labor was uneventful, the head was engaged, there was no significant antenatal bleeding, which is commonly present with annular placenta and the woman was delivered vaginally (1,3). Cases of annular placenta have been occasionally reported but the last report was nearly 20 years ago (2,4). We believe that reason for this is the rising incidence of cesarean section, in which the placenta is commonly damaged and some morphological conditions, including annular placenta, cannot be recognized. In this case, despite annular placenta, vaginal delivery occurred through the placental opening above the internal os, and annular placenta was recognized after delivery and confirmed on pathological examination.
